# Inhibitor in Congenital Factor VII Deficiency; a Rare but Serious Therapeutic Challenge—A Systematic Literature Review

**DOI:** 10.3390/jcm10020211

**Published:** 2021-01-08

**Authors:** Nahid Ramezanpour, Farhad Zaker, Arijit Biswas, Akbar Dorgalaleh

**Affiliations:** 1 Department of Hematology and Blood Transfusion, School of Allied Medicine, Iran University of Medical Sciences, Tehran 1449614535, Iran; n.ramezanpour.k@gmail.com (N.R.); zaker.f@iums.ac.ir (F.Z.); 2 Institute of Experimental Hematology and Transfusion Medicine, University Hospital of Bonn, Sigmund Freud Street 25, 53127 Bonn, Germany; Arijit.biswas@ukb.uni-bonn.de

**Keywords:** factor VII deficiency, inhibitor, intracranial hemorrhage, replacement therapy

## Abstract

Background: Congenital factor (F) VII deficiency is a rare coagulation factor deficiency with an estimated incidence of 1 per 500,000 individuals. Patients with severe FVII deficiency present a broad range of clinical presentations. Alloimmunization against exogenous FVII, as the main challenge of replacement therapy, is an extremely rare phenomenon that is accompanied by a high rate of life-threatening bleeding, that renders replacement therapy less effective. Due to the importance of the issue, we performed a systematic literature review in order to assess incidence, molecular basis, clinical presentations, and therapeutic challenge and management of inhibitor in congenital FVII deficiency. Strategy of search: This systematic review was performed in accordance with PRISMA guidelines. We performed an English-language literature review in the PubMed, EMBASE, Scopus, and Google Scholar databases, using the following keywords: “factor VII inhibitor”, “factor VII inhibitors”, “FVII inhibitors”, “congenital FVII deficiency”, “recombinant factor VII”, “anti rFVIIa”, “replacement therapy”, and “alloantibody”. Results: Out of 380 patients in the 13 studies, 27 had inhibitor against FVII; 18 were male, 7 were female, while the sex of 2 was not stated. The majority (92%) developed a high-titer inhibitor (Bethesda Unit > 5). All patients had severe FVII deficiency (FVII:C < 10%), and the majority received recombinant FVII prior to inhibitor development (N: 24, 89%). Among ten patients with a detected mutation, three subjects had a common non-sense (30%), and two had a deletion (20%). Conclusions: Inhibitor development is a relatively rare phenomenon seen only in severe FVII deficiency, where it is associated with severe and life-threatening presentations, treatment challenge, and economic burden on the patients and their families.

## 1. Introduction

Coagulation factor (F) VII is produced by the liver, circulating in the plasma in two forms, mainly as an inactive single chain. It interacts with tissue factor (TF) to initiate the coagulation cascade, more specifically, extrinsic pathway [1,2]. Deficiency of FVII, a rare coagulation factor deficiency (RCFD) is estimated to occur in 1 per 500,000 persons. Incidence of the other RCFDs—fibrinogen, FII (Prothrombin), FV, combined FV and FVIII (F5F8), FX, FXI, FXIII, and vitamin-K-dependent coagulation factor (VKCF)—varies from 1 per 500,000 to 1 per 2,000,000 [2,3]. FVII deficiency, like other autosomal recessive disorders, is more common in societies with a high rate of consanguineous marriages and can be classified, based on FVII coagulation activity (FVII:C), as mild (FVII:C > 20%), moderate (FVII:C 10–20%), or severe (FVII:C < 10%) [4]. Most commonly, FVII deficiency presents with epistaxis, hematoma, intracerebral hemorrhage (ICH), postsurgical bleeding, hemarthrosis, etc. [5].

The mainstay of treatment for congenital FVII deficiency is replacement therapy (RT), available as prothrombin complex concentrates (PCC), fresh frozen plasma (FFP), plasma-derived (pd)-FVII, and recombinant FVII (rFVIIa) [2]. However, RT carries the risk of alloantibody development, which decreases the treatment’s efficacy, requiring more complicated regimens, increases the cost of patient care, and may render the patient disabled [4,6]. Inhibitor, a very serious and life-endangering complication of RT, is an extremely rare phenomenon in FVII deficiency [7]. Due to the paucity of data, as well as the importance of the issue, in the present systematic literature review we aimed to assess incidence, clinical consequences, and management of inhibitor development in patients with congenital FVII deficiency, in order to create a primary scientific structure for further studies in the future.

## 2. Strategy of Search

This systematic literature review is performed in accordance with PRISMA guidelines. The inclusion criteria and search strategy for study selection were specified in advance. To this end, we analyzed the available literature on congenital FVII deficiency in patients with FVII inhibitor, focusing mainly on different aspects of inhibitor development including incidence, molecular characteristics, clinical presentations, and therapeutic strategy.

A comprehensive English-language search was conducted to identify all published studies (1990 up to the search cut-off date of 1 September 2020) related to our keywords, using the PubMed, EMBASE, Scopus, and Google Scholar electronic databases. Special attention has been paid to the reference list of some of the retrieved items, including eligible articles and recent reviews, to determine existence of additional publications. Our searches used the following keywords: “factor VII inhibitor”, “factor VII inhibitors”, “FVII inhibitors”, “congenital FVII deficiency”, “recombinant factor VII”, “anti rFVIIa”, “replacement therapy”, and “alloantibody”. Keywords were used separately and in combination with each other.

## 3. Study Selection and Inclusion Criteria

Two-level screening by two of the authors reviewed all non-duplicate citations to evaluate their eligibility for inclusion. Level I, performed by a single reviewer, examined the title and abstract of each article. In Level II, the full text of each citation, evaluated by a single reviewer, then was reviewed by another reviewer. Conflicts or disagreements between the reviewers’ opinions were further analyzed after a thorough discussion on the full text of the source. Thus, a citation was included in our study if both reviewers were in agreement. Selected studies met the following criteria: Only studies on human samples with congenital FVII deficiency were considered; the included studies had a minimum sample size of one patient; the study reported at least one exposure to replacement therapy, especially with rFVIIa. No restrictions were placed on age, sex, ethnicity, or type of replacement therapy.

## 4. Study Selection

**Figure 1:**
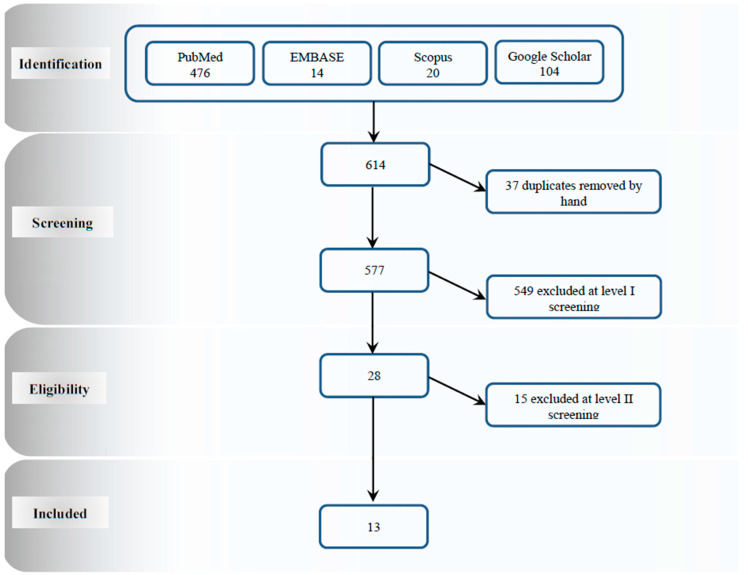
Study characteristics and selection.

As seen in the flowchart (Figure 1) our search in the mentioned four databases yielded a total of 614 studies. After removing 37 studies, due to duplications, from the list, 577 unique articles were identified, 28 of which were selected following title and abstract screening. Based on our inclusion criteria, 13 studies were found eligible after screening the full texts. Primary reasons for exclusion were: Acquired FVII deficiency, studies on animals, and no exposure to RT.

## 5. Results

### 5.1. Study and Patient Characteristics

In the 13 selected studies, 380 patients were identified to have congenital FVII deficiency; among them, 27 were reported to have developed FVII inhibitor. The main characteristics of the patients, including sex, age of study entry, age at deficiency diagnosis, age at inhibitor development, bleeding events, pre/post inhibitor treatment, parental consanguinity, genetics, and antibody titer (Bethesda unit (BU)), are reported in Table 1, Table 2, Table 3, Table 4 and Table 5. All patients with available coagulation FVII levels (N: 26, 100%) had been recognized to have severe congenital FVII deficiency (FVII:C < 10%). Furthermore, 37% (N:10 out of 27) of the patients only received rFVIIa prior to inhibitor development. Similarly, before the inhibitor formation, 7% (N:2 out of 27) of the patients were solely under the treatment with FFP, and 4% (N:1 out of 27) only received pdFVII. 37% (N:10 out of 27) of the patients had the history of receiving both rFVIIa and FFP prior to inhibitor development. For 11% (N:3 out of 27) of the patients had the experience of receiving both rFVIIa and pdFVII as the treatment in their medical history. There was just one patient (N:1 out of 27, 4%) who was under the treatment with all of the three treatments in her medical background. There were 5 (N:5 out of 27, 19%) patients (cases # 1, 18, 21, 25, and 26) who were reported to succumbed to the disease.

Among 27 cases, the age of inhibitor diagnosis was available for 22, among them 73% (N: 16) inhibitor was detected were under the age of five. For four patients, a parental consanguineous marriage had been reported in the literature. In this regard, cases #13 and #18 had a positive history, while cases #14 and #19 had a negative. More than 90% of patients (N: 23 out of 25) had a high-titer inhibitor (BU > 5). The citations identified 18 males and 7 females; one study gave no information about the sex of two patients [8]. Considering the studies in which the age of inhibitor formation has been reported, 5 patients were identified to have developed inhibitor under the age of six months.

Thirty-seven percent (N:10 out of 27) of patients received only rFVIIa prior to inhibitor development. Similarly, before inhibitor formation, 7% (N:2 out of 27) of patients were solely under treatment with FFP, and 4% (N:1 out of 27) received only pdFVII. Thirty-seven percent (N:10 out of 27) of patients had received both rFVIIa and FFP prior to inhibitor development. Eleven percent (N:3 out of 27) had the experience of receiving both rFVIIa and pdFVII as treatment. Just one patient (N:1 out of 27, 4%) was treated with all three substances. Five (N:5 out of 27, 19%) patients (cases # 1, 18, 21, 25, and 26) were reported to have succumbed to the disease.

### 5.2. Incidence of FVII Inhibitor in Congenital FVII Deficiency

Three studies were performed on the incidence of FVII inhibitors [4,6,9]. The first detailed study is the research of Batorova et al. [6], in the international Seven Treatment Evaluation Registry (STER), with an incidence rate of 2.6% (3 of 115 patients) [6]. The second, performed on 50 Iranian patients by Shams et al. [9], reported an incidence rate of 4% (2 of 50 patients). The most recent study, also conducted on Iranian patients, reported an 8.8% incidence rate (8 of 91 patients) [9]. Compared to previous research, the study of Eshghi et al. [9], has shown a significant increase in the rate of incidence.

### 5.3. Characterization of the FVII Inhibitor

Regarding the nature and characterization of the FVII inhibitor, we found three research papers in the literature [10,11,12]. Using multiplex assay, Tokgoz et al., [10] assessed the reactivity of antibody, total IgG, and IgG subclasses against rFVIIa. It showed that the alloimmune response was polyclonal and heterogeneous (various IgG subclasses involved); a predominant T helper 2 role in the antibody response to the FVII antigen has been suggested due to the high specificity of the subclass IgG4 [10].

The second study, Branchini et al. [11], confirmed that the immune response was polyclonal; there was a notable predominance of the IgG1 subclass. Another finding of this research was the higher affinity of antibodies toward the active rather than zymogen form of FVII [11].

The third study researched the nature of FVII inhibitor, using an immunoassay based on the x-MAP technology to analyze the isotype of the antibodies against rFVIIa [12]. According to the study’s findings, the high and low specificity of the subclasses IgG1 and IgG3, respectively, suggests a predominant T helper 1 contribution in the alloimmune response. It showed that this response also was polyclonal. It mentioned that, in contrast to their patient, the patient with an IgG4 subclass in the research of Tokgoz et al. [10], has shown a successful response to rFVIIa [10]. All three studies were agreed on the polyclonal nature of the immune system’s response, but only two reported a large predominance of the IgG1.

### 5.4. Molecular Basis

Among the FVII inhibitor-related studies reported to date, mutations have been reported in 10 out of 27 patients. Notably, no mutation in the coding regions of FVII, or large rearrangement, could be identified in case #13. According to Table 1, Table 2, Table 3 and Table 4, four cases have missense mutations in F7 gene (#3, #4, #14, and #19) (100 GLN → ARG shift, Ser103 → Gly (S103G), IVS6 + 1G > T, and c.572–12T > A, respectively). Three patients (#5, #10, and #15) have nonsense mutations (p.Ser112-Stop). Patient #9 has a double missense mutation and a single deletion (p.A354V-p.P464Hfs). Two patients (#24 and #26) have c.9711 deletion on exon 7.

**Table 1:** Characteristics of 27 FVIID patients with FVII inhibitor (case #1 to #7).

Author	Nicolaisen et al. (1996) [8]	Nicolaisen et al. (1996) [8]	Ingerslev et al. (2005) [13]	Pruthi et al. (2007) [14]	Tokgoz et al. (2012) [10]	Mariani et al. (2013) [5]	Mariani et al. (2013) [5]
	Case #1	Case #2	Case #3	Case #4	Case #5	Case #6	Case #7
Sex	N/A	N/A	Female	Male	Male	Male	Female
1st age *	N/A	N/A	N/A	11 days	3 months	N/A	N/A
2nd age *	N/A	N/A	~36 years	45 years	1 year	30 days after RT	30 days after RT
3rd age *	2 months	N/A	39 years	51 years	3 months	1 year	54 years
FVII:C	<4%	N/A	<0.01%	<1%	<1%	2.50%	<1%
Bleeding events	ICH	N/A	MSB	Hr, Ep	ICH, GIB	CNS, Br, Ep, GuB, hematomas	Br, Ep, GuB, HmB, Me
Genetics	N/A	N/A	Homozygosity for a nucleotide substitution that caused 100 GLN→ ARG shift	Missense mutation in F7 gene Ser103→Gly (S103G)	Homozygous nonsense mutation in F7 p.Ser112-Stop	N/A	N/A
Antibody titers (BU) min-max	N/A	N/A	High titer inhibitors for both pdFVII (16 BU/mL) and rFVIIa (88 BU/mL).	Less than 1 to 4	34–68.3	2.1	22.4

* 1st age: Age at congenital FVIID diagnosis, 2nd age: Age at inhibitor development, and 3rd age: Age of study entry. The history of parental consanguineous marriage had been reported for four cases among the above 27 patients. In this regard, cases #13 and #18 were positive and cases #14 and #19 were negative. N/A: Not available, BU: Bethesda unit, RT: Replacement therapy, FVII:C: Factor VII coagulant activity, SDH: Subdural hemorrhage, IVH: Intraventricular hemorrhage, ICH: Intracerebral hemorrhage, GIB: Gastrointestinal bleeding, MSB: Musculoskeletal bleeding, Hr: Hemarthrosis, Ep: Epistaxis, CNS: Central nervous system, Br: Easy bruising, GuB: Gum bleeding, He: Hematuria, Me: Menorrhagia, HmB: Hemorrhoidal bleeding, Hp: Hemoperitoneum, pdFVII: Plasma derived FVII, and rFVII: Recombinant FVII. “→” means a shift in a amino acid.

**Table 2:** Characteristics of 27 FVIID patients with FVII inhibitor (case #8 to #14).

Author	Napolitano et al. (2013) [15]	Batorova et al. (2014) [6]	Batorova et al. (2014) [6]	Batorova et al. (2014) [6]	Batorova et al. (2014) [6]	Borhany et al. (2015) [12]	See et al. (2016) [2]
	Case #8	Case #9	Case #10	Case #11	Case #12	Case #13	Case #14
Sex	Male	Female	Male	Male	Male	Female	Female
1st age *	N/A	N/A	N/A	N/A	N/A	N/A	Prenatal period
2nd age *	1 year after FFP	59 years	3 months	5 months	1 month	N/A	4 years
3rd age *	5 years	53 years	5 years	5 months	1 year	8 years	5 years
FVII:C	2%	<1%	2%	1.30%	<1%	1%	<1%
Bleeding events	CNS, Br, Ep, GIB, GuB	Ep, Br, GuB, Hp, Me	CNS, Br, Ep, GIB, GuB	CNS, GIB, GuB	CNS, Br, Ep, GuB, hematomas	Hematoma, Hr	SDH, IVH, ICH, Hr
Genetics	Homozygous (gene mutations were only reported as zygosity)	p.A354V-p.P464Hfs	p.Ser112-Stop (homozygous)	N/A	N/A	No Identified mutation in the *F7* coding regions and large rearrangement	Homozygous mutation in intron 6 of the *F7* gene IVS6 + 1G > T (c.681 + 1G > T)
Antibody titers (BU) min-max	59	10–20	38–68.3	5.5–60	32–72	1.1–11	2.77–20.6

* 1st age: Age at congenital FVIID diagnosis, 2nd age: Age at inhibitor development, and 3rd age: Age of study entry. The history of parental consanguineous marriage had been reported for four cases among the above 27 patients. In this regard, cases #13 and #18 were positive and cases #14 and #19 were negative. N/A: Not available, BU: Bethesda unit, RT: Replacement therapy, FVII:C: Factor VII coagulant activity, SDH: Subdural hemorrhage, IVH: Intraventricular hemorrhage, ICH: Intracerebral hemorrhage, GIB: Gastrointestinal bleeding, MSB: Musculoskeletal bleeding, Hr: Hemarthrosis, Ep: Epistaxis, CNS: Central nervous system, Br: Easy bruising, GuB: Gum bleeding, He: Hematuria, Me: Menorrhagia, HmB: Hemorrhoidal bleeding, Hp: Hemoperitoneum, pdFVII: Plasma derived FVII, and rFVII: Recombinant FVII. “→” means a shift in a amino acid.

**Table 3:** Characteristics of 27 FVIID patients with FVII inhibitor (case #15 to #21).

Author	Tokgoz et al. (2017) [16]	Tokgoz et al. (2017) [16]	Shams et al. (2018) [4]	Shams et al. (2018) [4]	Patel et al. (2019) [17]	Eshghi et al. (2019) [9]	Eshghi et al. (2019) [9]
	Case #15	Case #16	Case #17	Case #18	Case #19	Case #20	Case #21
Sex	Male	Male	Male	Male	Male	Female	Female
1st age *	N/A	N/A	11 days	60 years	N/A	4 days	3 years
2nd age *	1 year	<6 months	7 years	66 years	7 months	2.5 years	~3 years
3rd age *	3 months	11 days	14 years	70 years	3 months	N/A	N/A
FVII:C	0.20%	0.10%	<1%	6%	<1%	<10%	<10%
Bleeding events	ICH	Ep, ICH	Hematoma, ICH, Hr, Ep, Br	Br	ICH	Hematoma, GIB, ICH, Ecchymosis, He, Ep	Hematoma, ICH, He, Hr
Genetics	homozygous nonsense mutation in *F7* gene, p.Ser112stop	N/A	N/A	N/A	Homozygous mutation in *F7* gene Intron 5, c.572–12T>A	N/A	N/A
Antibody titers (BU) min-max	32	28.8	5–170	5–10	2.2–819	7–34.4	80

* 1st age: Age at congenital FVIID diagnosis, 2nd age: Age at inhibitor development, and 3rd age: Age of study entry. The history of parental consanguineous marriage had been reported for four cases among the above 27 patients. In this regard, cases #13 and #18 were positive and cases #14 and #19 were negative. N/A: Not available, BU: Bethesda unit, RT: Replacement therapy, FVII:C: Factor VII coagulant activity, SDH: Subdural hemorrhage, IVH: Intraventricular hemorrhage, ICH: Intracerebral hemorrhage, GIB: Gastrointestinal bleeding, MSB: Musculoskeletal bleeding, Hr: Hemarthrosis, Ep: Epistaxis, CNS: Central nervous system, Br: Easy bruising, GuB: Gum bleeding, He: Hematuria, Me: Menorrhagia, HmB: Hemorrhoidal bleeding, Hp: Hemoperitoneum, pdFVII: Plasma derived FVII, and rFVII: Recombinant FVII. “→” means a shift in a amino acid.

**Table 4:** Characteristics of 27 FVIID patients with FVII inhibitor (case #22 to #27).

Author	Eshghi et al. (2019) [9]	Eshghi et al. (2019) [9]	Eshghi et al. (2019) [9]	Eshghi et al. (2019) [9]	Eshghi et al. (2019) [9]	Eshghi et al. (2019) [9]
	Case #22	Case #23	Case #24	Case #25	Case #26	Case #27
Sex	Male	Male	Male	Male	Male	Male
1st age *	2 months	2 months	2 weeks	16 days	2 months	2 months
2nd age *	2 years	2 years	8 years	3 months	2 years	~3 years
3rd age *	N/A	N/A	N/A	N/A	N/A	N/A
FVII:C	<10%	<10%	<10%	<10%	<10%	<10%
Bleeding events	Hematoma, GIB, ICH, Ecchymosis	Hematoma, GIB, ICH, Hr, Ecchymosis	GIB, ICH, Hr, Mucocutaneous, Ep	ICH	ICH	ICH Mucocutaneous, Ecchymoses, Ep
Genetics	N/A	N/A	c.9711 deletion on exon 7	N/A	c.9711 deletion on exon 7	N/A
Antibody titer (BU) min-max	5–36	43–217	0–170	14.7	191	7

* 1st age: Age at congenital FVIID diagnosis, 2nd age: Age at inhibitor development, and 3rd age: Age of study entry. The history of parental consanguineous marriage had been reported for four cases among the above 27 patients. In this regard, cases #13 and #18 were positive and cases #14 and #19 were negative. N/A: Not available, BU: Bethesda unit, RT: Replacement therapy, FVII:C: Factor VII coagulant activity, SDH: Subdural hemorrhage, IVH: Intraventricular hemorrhage, ICH: Intracerebral hemorrhage, GIB: Gastrointestinal bleeding, MSB: Musculoskeletal bleeding, Hr: Hemarthrosis, Ep: Epistaxis, CNS: Central nervous system, Br: Easy bruising, GuB: Gum bleeding, He: Hematuria, Me: Menorrhagia, HmB: Hemorrhoidal bleeding, Hp: Hemoperitoneum, pdFVII: Plasma derived FVII, and rFVII: Recombinant FVII. “→” means a shift in a amino acid.

### 5.5. Clinical Manifestations

Among 27 patients with FVII deficiency and inhibitor, addressed in our systematic review (presented in Table 1, Table 2, Table 3 and Table 4), clinical manifestations had been reported for all cases but one (#2). All 26 patients had FVII:C levels below 10%. About 58% of patients (N: 15) had ICH, 46% experienced epistaxis, 35% had the hematoma phenotype, and 31% showed symptoms of gastrointestinal bleeding. An equal percentage of patients (27%) experienced hemarthrosis or gum bleeding. There was ecchymosis in 15% of the patients, and 8% had hematuria. Among females (7 out of 25—the sexes of two patients (#1 and #2) were not available), two (nearly 28%) reported menorrhagia.

### 5.6. Management of Inhibitor

According to some case reports and case series, the use of rFVIIa can be either successful or unsuccessful in managing patients regardless of the way it is used (repetitive or single high-dose) [9,13]. Regarding the patients addressed in this study (Table 1, Table 2, Table 3, Table 4 and Table 5), 12 out of 27 (44%) cases were explicitly reported to have been successfully managed with rFVIIa after inhibitor formation (cases 5, 9, 10–12, 14, 15, 16, 20, 22, 23, and 27). Cases 1, 13, 18, 21, and 24–26, however, were not responsive to rFVIIa. Three patients (cases 21, 25, and 26), treated with a high dose of rFVIIa or FEIBA (Factor Eight Inhibitor Bypassing Activity), died due to recurring ICH. Cases 1 and 18, who received only rFVIIa, also died. Case 13 was not responsive to rFVIIa, nor to extensive subsequent immunosuppressive treatment. In case 24, although the patient was unresponsive to rFVIIa (NovoSeven^®^), the treatment was followed by FEIBA (80–100 IU/kg). An immune tolerance induction (ITI) regimen, pd-FVII concentrate was successfully used to control the inhibitor for case 19. Post-inhibitor treatment with FEIBA elicited excellent clinical response in case 17. Details of the treatment can be found in Table 5.

**Table 5:** Treatments and outcomes of patients with FVII inhibitor.

Case Number	Author	Pre-Inhibitor Treatment	Post-Inhibitor Treatment	Outcome
#1	Nicolaisen et al. (1996) [8]	FFP, rFVIIa (approximately 800 μg/kg)	N/A	High-dose of rFVIIa (800 Ig/kg) was used following no ICH improvement with FFP. Although it elicited a good initial response, the treatment failed, and the patient died.
#2	Nicolaisen et al. (1996) [8]	pd-FVII, rFVIIa	N/A	While there was no antibody detected after exposure to plasma-derived FVII, the patient developed inhibitor three months after being exposed to rFVIIa.
#3	Ingerslev et al. (2005) [13]	pd-FVII, rFVIIa (161 mg for 6 years)	rFVIIa (20 μg/kg/body weight)	The patient developed an inhibitor after exposure to pd-FVII (16 BU/mL) and rFVIIa (88 BU/mL). The inhibitor titer later was found to have decreased due to cessation of treatment with FVII-containing material. Re-exposure to 1.2 mg rFVIIa has led to an anamnesis with a notable increase in inhibitor titers.
#4	Pruthi et al. (2007) [14]	FFP	The inhibitor resolved spontaneously after two years	Four months after FFP exposure, a low-titer FVII inhibitor was detected, which resolved spontaneously in the absence of further FFP infusions. Inhibitor was detected again after renewed exposure to FFP and rFVIIa.
#5	Tokgoz et al. (2012) [10]	rFVIIa (20–30 μg/kg), FFP (20 ml/kg per week)	rFVIIa (20–30 μg/kg), prophylaxis treatment initiated at 30 Ig/kg 3 × per week at the age of 4.5	A successful prophylactic rFVIIa (30 µg/kg, three times per week) was reported in a 4.5-year-old patient who had congenital FVIID, with inhibitors to FVII.
#6	Mariani et al. (2013) [5]	rFVIIa (65 μg/kg)	N/A	The patient’s bleeding episodes were treated using plasma-derived FVII (with a total dose of 33 µg/kg) in a repetitive procedure.
#7	Mariani et al. (2013) [5]	Pd-FVII (33 μg/kg)	N/A	The patient’s bleeding episodes were treated using rFVIIa (with the total dose of 65 µg/kg) in a repetitive procedure.
#8	Napol-tano et al. (2013) [15]	FFP	rFVIIa (90 total weekly dose mg/kg/body weight, 3×weekly)	After being treated with FFP, this patient developed FVII inhibitor (max titer: 59 BU). Because of recurrent CNS bleeding prophylactic rFVIIa was initiated.
#9	Batorova et al. (2014) [6]	FFP, pd-FVII, rFVIIa	rFVIIa (Initial bolus 30 μg/kg plus 8 consecutive boluses 10 μg/kg)	Congenital FVII deficiency was diagnosed following epistaxis in a patient. Treatments with FFP, pd-FVII and rFVIIa were scheduled. At the age of 59 years and during multiple dental extractions, FVII inhibitor diagnosed and treated by rFVIIa.
#10	Batorova et al. (2014) [6]	FFP, rFVIIa	rFVIIa (30 μg/kg × 3 weeks)	FFP was given to a 3-month-old patient after CNS bleeding. He was also given prophylactic rFVIIa. Despite the development of FVII inhibitor at this age, he was treated by rFVIIa.
#11	Batorova et al. (2014) [6]	FFP, rFVIIa	rFVIIa (65 μg/kg × 1 weeks)	Following CNS and GI bleeding at birth, FFP was administered. He was then given prophylactic rFVIIa as well. Despite the development of FVII inhibitor at the age of 5 months, he was treated with rFVIIa.
#12	Batorova et al. (2014) [6]	FFP, rFVIIa	rFVIIa (31 μg/kg × 3 weeks)	Following tongue hematoma, FFP was scheduled for a one-month-old patient. This was followed by prophylactic rFVIIa (31 µg/kg × 3 week). Despite the development of FVII inhibitor at this age, he was treated with rFVIIa.
#13	Borhany et al. (2015) [6]	rFVIIa (15 μg/kg	Steroid (azathioprine), Immunoglobulin, FEIBA	Hemarthrosis and gum bleeding were controlled by rFVIIa (15 lg/kg every 6 hours). However, some bleedings developed, with no response to rFVIIa two weeks after this treatment. Therefore, rFVIIa infusion stopped, and extensive immunosuppressive treatment was scheduled. Despite this, the patient’s clinical status deteriorated.
		then increased to 30 μg/kg), FFP (15 mL/kg), vitamin K injections		

#14	See et al. (2016) [2]	rFVIIa (30 μg/kg), rFVIIa (30 μg/kg), rFVIIa (30–90 μg/kg, depending on severity of bleeding), rFVIIa, vitamin K therapy	rFVIIa (30 μg/kg per dose), secondary prophylaxis with rFVIIa (30 μg/kg per dose twice a week)	This patient went through an auxiliary liver transplant. Twenty-two days after prophylactic rFVIIa injection, which was performed during the transplantation, the inhibitor titer reached a detectable level (20.6 Bethesda units). Despite this inhibitor development, the patient was still responsive to rFVIIa (30 Ig/kg).
#15	Tokgoz et al. (2017) [16]	rFVIIa	rFVIIa (30 μg/kg, three times a week) was used; increased to 30 μg/kg every day)	Inhibitor (32 BU) at the age of one. The same doses of rFVIIa were sufficient to control all bleeding episodes even in the presence of inhibitor. Although prophylactic rFVIIa was successful in preventing bleeding episodes for 4 years, this dose became ineffective afterward, hence prophylactic rFVIIa doses were increased.
#16	Tokgoz et al. (2017) [16]	rFVIIa (25 mcg/kg) then prophylaxis (25 mcg/kg, 3 times a week) initiated	40 mcg/kg rFVIIa was used; and followed by gradual increase of rFVIIa to 100mcg/kg/day	The ICH of this patient was treated with rFVIIa after two weeks. Then the patient went through prophylactic rFVIIa. Nevertheless, the patient developed FVII inhibitor (28.8 BU). After this development, the bleeding, however, was controlled after three weeks and with an increased dose of rFVIIa. The prophylactic rFVIIa was later gradually increased.
#17	Shams et al. (2018) [4]	rFVIIa	FEIBA	Administration of rFVIIa was successful in treatment of a 14-day-old boy. Three months later, rFVIIa was used for shunt replacement. Since the age of approximately 6 months, regular prophylaxis was scheduled for the patient until the age of seven. At age 10, inhibitor level was 5BU. Three years later, pre-operative rFVIIa notably increased the level of inhibitor titer (70 BU). Thereafter, the clinical response to on-demand therapy (FEIBA) was excellent.
#18	Shams et al. (2018) [4]	rFVIIa (50 μg/kg), then secondary prophylaxis initiated	rFVIIa	This patient was randomly diagnosed with congenital FVIID prior to angiography at age 60. The procedure was performed by rFVIIa. After 6 years, the inhibitor was detected and the patient went through several rFVIIa uses.
#19	Patel et al. (2019) [17]	rFVIIa (NovoSeven^™^), switched to pd-FVII (20 IU/kg daily, then x3/week)	Pd-FVII (50 IU/kg (rounded to 600 IU) of pd FVII concentrate x3/week, and 50 IU/kg (600IU) daily, rFVIIa (NovoSeven^™^)	This patient was identified with inhibitor to FVII at the age of 7. An ITI regime was used to control the production of antibody in the patient.

#20	Eshghi et al. (2019) [9]	rFVIIa (AryoSeven^™^) (50 μg/kg/day on alternate days), FFP	High dose AryoSeven^™^ (120 μg/kg/day)	The patient was responsive to high dose of AryoSeven^™^ (120 μg/kg/day). Afterwards, as a prophylactic treatment, the same type of rFVIIa was used up to 100 ug/kg every other day. Current clinical status of the patient is “no symptoms”.
#21	Eshghi et al. (2019) [9]	rFVIIa (AryoSeven^™^): 30 μg/kg/every other day, FFP	No response to high-dose AryoSeven^™^: 100 μg/kg, switch to FEIBA: 50–75 u/kg/day, three times a day	The patient was treated with FEIBA. Prophylactic treatment (75 ug/kg) was used three times a week. The patient succumbed to severe ICH.
#22	Eshghi et al. (2019) [9]	rFVIIa (NovoSeven^®^ and AryoSeven^™^): 30 μg/kg, 3 times a week	High-dose AryoSeven^™^: 45 μg/kg	This patient was responsive to high dose of AryoSeven^™^ (45 ug/kg/ twice per week). The current clinical status of the patient is “no symptoms”.
#23	Eshghi et al. (2019) [9]	rFVIIa(AryoSeven^™^): 30 μg/kg, 3 times a week, FFP	Blood transfusion, FEIBA for a short period, then high dose AryoSeven^™^: 40–45 μg/kg	A prophylactic treatment was used for this patient (AryoSeven^™^, 45 ug/kg, 3 times a week). No severe bleeding events were reported.
#24	Eshghi et al. (2019) [9]	rFVIIa (NovoSeven^®^): 50 μg/kg/day (on-demand), FFP	No response to NovoSeven^®^.FEIBA: 80 u/kg/twice a day	Following the treatment with FEIBA (80–100 IU/kg), inhibitor titer reduced to 20. Six months later the inhibitor level reached zero and the patient became responsive to on-demand rFVIIa.
#25	Eshghi et al. (2019) [9]	rFVIIa(AryoSeven^™^): 30 ug/kg, daily	High-dose AryoSeven^™^: 270 μg/kg FEIBA, FFP, Cryo	The patient was not responsive to high dose of AryoSeven^™^; the patient died due to extensive ICH.
#26	Eshghi et al. (2019) [9]	rFVIIa (NovoSeven^®^): Not applicable (on-demand)	No response to NovoSeven^®^.FEIBA: 75-80 u/kg twice per day	This patient, who was suffering from sepsis and intra-abdominal bleeding, was not responsive to NovoSeven^®^ and FFP. He died at age 4 due to sepsis followed by DIC.
#27	Eshghi et al. (2019) [9]	rFVIIa (AryoSeven^™^): 40 μg/kg/day on alternate days	High-dose AryoSeven^™^: 70 μg/kg/day	The patient was responsive to high dose of AryoSeven^™^. The current clinical status of the patient was reported to be “symptomatic”.

N/A: Not available, BU: Bethesda unit, ICH: Intracerebral hemorrhage, GI: Gastrointestinal, CNS: Central nervous system, pdFVII: Plasma derived FVII, rFVII: Recombinant FVII, FFP: Fresh frozen plasma, FVIID: Factor VII deficiency, FEIBA: Factor eight inhibitor bypassing activity, ITI: Immune tolerance induction and DIC: Disseminated intravascular coagulation.

## 6. Discussion

Regarding inhibitor development in congenital FVII deficiency, few data are available in the literature. The formation of inhibitor to a clotting factor increases the rate of morbidity and mortality, imposes social and spiritual burdens on patients and their families, increases the cost of care, and, in the end, more complications in treatment regimens [18,19]. When it comes to FVII replacement treatment, the development of inhibitory alloantibody against the factor is an important challenge. Patients with FVII deficiency and inhibitor developed a high rate of severe, life-threatening bleeding. About 60% experienced the most dreadful presentation, ICH, whereas the rate for patients without inhibitor is less than 10%. Other major bleeding episodes, such as hematoma, hemarthrosis, and gastrointestinal bleeding, were observed commonly. It seems that inhibitor development aggravates bleeding presentations in FVII deficiency, a finding that was observed in other rare- and common-coagulation factor deficiencies [3]. In addition, as with other factor deficiencies, inhibitor development makes treatment and management of bleeding more challenging, with higher incidence of treatment failure and subsequent death, as reported by Eshghi et al. [9]. Management of bleeding in patients with inhibitor is a significant and sophisticated process, the main goals of treatment being to stop bleeding, and to eradicate inhibitor [4]. In one study on Iranian patients, immunosuppression and eradication of inhibitor had acceptable results. However, the study also observed failure of the treatment, with fatal consequences [9]. The literature review also highlighted that almost all patients with inhibitor had severe FVII deficiency [6,13,14]. In these patients, exogenous infused factor is more likely to be identified as an antigen, leading to immune response and antibody formation. These patients also are more likely to receive RT, due to their higher rate of bleeding [12]. In other coagulation factor deficiencies, such as hemophilia A and B, the rate of inhibitor formation is significantly higher in patients with severe deficiency than mild and moderate deficiencies [20,21]. Similar to hemophilia, the type of replacement therapy, especially with rFVIIa, may play a major role in the complex, multifunctional process of inhibitor formation [11,22]. Although there is a similarity between the gene and protein structures of FVII and FIX, there are some notable differences. For instance, severe gene defects such as large deletion have been reported in patients with hemophilia B with inhibitors; there is no report of such defects in patients with FVII deficiency [23]. Unlike in FVII deficiency, moreover, the immune response in patients with hemophilia B could be complicated by anaphylactoid reaction, which is a severe clinical issue [6,24,25]. Studies conducted on the nature of FVII inhibitor agreed that the type of immune response was polyclonal, with two of the three reporting a predominance of the IgG1 subclass, whereas the third suggested subclass IgG4 [10,11,12].

In the current research we identified 13 studies published from 1990 to 2020 which focus primarily on FVII inhibitor. The aforementioned studies identified 380 patients, among whom 27 developed FVII inhibitor. The incidence rate of FVII inhibitor occurrence, in this group of patients, thus is approximately 7%, which is reasonably close to that reported by Eshghi et al. [9]. This rate of inhibitor formation seems to be higher than that in most RCFDs, but lower than hemophilia A and B [9,21].

Except for two patients (cases #1 and #2) whose sex is not mentioned, 7 patients were female and 18 patients were male; rates of FVII inhibitor occurrence were ~28% and 72%, respectively. The rate of inhibitor development in congenital FVII deficiency seems to be higher in males, but, due to the low number of patients, further studies are required to assess this finding. As can be seen in Table 1, Table 2, Table 3 and Table 4, the genotype of 10 of the 27 patients has been reported in the literature to date. Forty percent of patients had missense mutations (#3, #4, #14, and #20), 30% (#5, #10, and #16) had nonsense mutations, 20% (#21 and #23) had c.9711 deletion on exon 7, and #9 (10%) had a double missense mutation and a single deletion. Although genetic factors might have a role in inhibitor development in FVII deficiency, such a conclusion cannot be reached from available reports due to the small size of the study population. In hemophilia A, genetic risk factors play an important role in inhibitor formation; large deletions, nonsense mutations, and some polymorphisms make the patients more prone to this phenomenon [26,27,28].

Although relatively rare, inhibitor can be formed in congenital FVII deficiency, with life-threatening bleeding, presenting a significant therapeutic challenge which may have fatal consequences. Familiarity with different aspects of inhibitor could help to prevent inhibitor formation and manage this life-threatening condition.
